# CT pulmonary angiography in COVID-19 pneumonia: relationship between pulmonary embolism and disease severity

**DOI:** 10.1186/s43055-020-00389-7

**Published:** 2021-01-05

**Authors:** Aya Yassin, Maryam Ali Abdelkader, Rehab M. Mohammed, Ahmed M. Osman

**Affiliations:** 1 Radiology, Faculty of Medicine, Ain Shams University, Cairo, Egypt; 2 Chest Diseases, Faculty of Medicine, Ain Shams University, Cairo, Egypt

**Keywords:** CT pulmonary angiography (CTPA), Pulmonary embolism (PE), D-dimer, COVID-19

## Abstract

**Background:**

Pulmonary embolism (PE) is one of the known sequels of COVID-19 infection. We aimed to assess the incidence of PE in patients with COVID-19 infection and to evaluate the relationship between the CT severity of the disease and the laboratory indicators. This was a retrospective study conducted on 96 patients with COVID-19 infection proved by positive PCR who underwent CT pulmonary angiography (CTPA) with a calculation of the CT severity of COVID-19 infection. Available patients’ complaint and laboratory data at the time of CTPA were correlated with PE presence and disease severity.

**Results:**

Forty patients (41.7%) showed positive PE with the median time for the incidence of PE which was 12 days after onset of the disease. No significant correlation was found between the incidence of PE and the patients’ age, sex, laboratory results, and the CT severity of COVID-19. A statistically significant relation was found between the incidence of PE and the patients’ desaturation, hemoptysis, and chest pain. A highly significant correlation was found between the incidence of PE and the rising in the D-dimer level as well as the progressive CT findings when compared to the previous one.

**Conclusion:**

CT progression and the rising in D-dimer level are considered the most important parameters suggesting underlying PE in patients with positive COVID-19 infection which is commonly seen during the second week of infection and alert the use of CT pulmonary angiography to exclude or confirm PE. This is may help in improving the management of COVID-19 infection.

## Background

COVID-19 pandemic outbreak started in Egypt in February 2020 and is still going on. The Egyptian protocol includes doing a non-contrast CT chest for patients with suspicious respiratory symptoms. However, many patients with worsened symptoms especially during the second week of disease showed symptoms of thrombotic events of pulmonary arteries, and that is why CT pulmonary angiography (CTPA) became recommended by multiple reports [1].

Association with elevated levels of D-dimer has been found in many patients with pulmonary embolism especially severely ill patients in intensive care units (ICU). Thromboprophylaxis is recommended for those patients with worsened symptoms and elevated laboratory indicators of coagulopathy [2].

Many reports emerged in the literature showing an increased incidence of a pulmonary embolism (PE) at segmental and subsegmental levels. The percentage was 30% in patients with COVID-19 pneumonia as compared to 1% in critically ill patients without COVID-19 disease [3].

Another study showed that 23% of CTPA performed at their institution were positive for PE. Those were diagnosed on average 12 days after onset of symptoms and most of them required ICU admission [4].

Most studies showed that patients with high body mass index (BMI) are more prone to embolic events. Those studies were trying to link thrombotic events to the severity of the disease, elevated D-dimer, and patient weight and associated diseases rather than a complication of hospitalization [5].

CT pulmonary angiography not only can assess the presence of pulmonary embolus but also can assess the severity of the embolus as well as heart function and strain on the right ventricle. Thus, we can predict the need for ICU admission and patient outcome [6].

This study aimed to assess the presence of PE in patients with COVID-19 infection with severe respiratory symptoms and to evaluate the relation to disease severity on CT as well as laboratory indicators as D-dimer, CRP, ferritin, and lymphocyte count. This is may help in more understanding, diagnosis, and management of COVID-19 infection.

## Methods

This was a retrospective study that was approved in our institutional ethical review board with the written consent waived. The authors had access to the data with no conflict of interest to declare. The study was conducted from March 1 till July 31.

### Patients

The study was conducted on 96 patients at one of the University Hospitals in Cairo, Egypt.

#### Inclusion criteria

All patients with COVID-19 infection proved by positive PCR who underwent CT pulmonary angiography. No age or sex predilection. Available patients’ complaints and laboratory data at the time of CTPA scanning.

#### Exclusion criteria

We excluded patients with unavailable PCR results, as well as patients with no data about the clinical condition and unavailable laboratory results.

### CT technique

- *Patients’ preparation*: Patients were required to fast for 4 h, and normal kidney function was needed using serum creatinine as a reference. Adequate hydration was instructed before and after the procedure. An 18–20-gauge cannula was inserted into the antecubital vein.
- *CT machine*: 80-slice CT machine (Prime Aquilion, Toshiba, USA) and Dual-source CT (Somatom Definition, Siemens Medical Solutions, Forchheim, Germany). The infection control parameters were applied under the guidance of the hospital infection control unit.
- The patients were scanned in a supine position with the arm above the head. A breath-hold was requested from the patients trying to avoid respiratory motion artifact. A region over interest was drawn on the main pulmonary artery. Bolus IV injection of nonionic contrast medium (Ultravist 370) 1.2 ml/kg was used at rate 4 ml/s using injector pump (Medrad injector) followed by 40 ml saline at rate 4 ml/s.
- *CT parameters*: The scan area extended from diaphragm to lung apex with scan time used = 10 s. The tube voltage was 140/80 KVP and tube current: 51/213 mAs. Rotation time was 0.33 s, 0.75 mm thickness, 0.7 reconstruction increment (mm), and 0.7 Pitch.
- *Image processing and interpretation*: The images were transferred to the workstation where the axial cuts and multi-planar reformation were reviewed by two radiologists experienced in chest imaging with at least 5 years of experience. They were blinded to the aim of the study and the clinical condition of the patients or the laboratory results. The following items were fulfilled:
  - Presence or absence of pulmonary embolism. If positive pulmonary embolism, site if unilateral or bilateral and extent if segmental, lobar, or main arterial.
  - CT probability of COVD-19 infection according to RSNA recommendations [7].
  - CT severity score for COVID-19 infection: The lung was divided into five lobes and was visually scored on a scale of 0 to 5. Score 0 if no CT evidence of pulmonary involvement; score 1 if the pulmonary involvement was less than 5%; score 2 if 5–25%; score 3 if 26–49%; score 4 if 50–75%; and score 5 if more than 75%. The highest total CT score was 25.
  - Presence or absence of CT progression comparing the CTPA with the last available study regarding the parenchymal involvement.

*The patients were divided according to the CTPA results into two groups: a group with positive PE and a group with negative PE.*

The clinical and laboratory data included the following:

- The timing of the CTPA after the onset of the symptoms.
- The main complaint at the time of CTPA scanning.
- The laboratory results including the WBC count, neutrophil count, lymphocyte count, D-dimer level, ferritin, and CRP levels. All laboratory results included must be within 24 h from the time of CTPA.

### Sample size calculation

Using PASS 11 program for sample size calculation, confidence level 95%, and a margin of error ± 0.1 and by reviewing previous study results by Grillet et al. [4] showed the rate of pulmonary embolism among COVID-19-positive patients (23%); based on that, the required sample size will be 76 patients with COVID-19 to be sufficient to estimate the rate of pulmonary embolism in COVID-19 patients.

### Statistical methods

All the data were collected, compared, and analyzed using IBM SPSS statistics (V. 26.0, IBM Corp., USA, 2019) for data analysis. Data were expressed as mean ± SD for quantitative parametric measures in addition to median and percentiles for quantitative nonparametric measures and both number and percentage for categorized data. The following tests were done:

1. Comparison between two independent groups for non-parametric data using the Wilcoxon rank-sum test.
2. Chi-square test to study the association between every 2 variables or comparison between 2 independent groups as regards the categorized data. The probability of error at 0.05 was considered significant, while at 0.01 and 0.001 are highly significant.

## Results

The study included 96 patients proved by PCR to have COVID-19 infection. Forty patients representing 41.7% of the patients showed positive PE. The mean age of the patients was 47.3 ± 19.9 years with age range 5–74. Fifty-eight patients were males while the rest 38 were female patients. No significant difference was found between the incidence of pulmonary embolism and the patients’ age and sex. The main complaint at the time of CTPA was progressive dyspnea detected in 64 patients (66.7%). Although progressive dyspnea was the highest in incidence regarding the complaint at the time of CTPA scanning, yet it showed no significant relationship with the incidence of PE. However, oxygen desaturation, chest pain, and hemoptysis showed a statistically significant relationship with the incidence of PE being significantly higher between patients with positive PE (Table 1).

**Table 1:** Illustrate the incidence and the relation between the patients’ sex, main complaint at the time of CTPA scanning, rising D-dimer level, CT interpretation according to RSNA criteria, and the progressive CT findings between the negative and positive pulmonary embolism groups under study

		***Negative CT pulmonary angiography***	***Positive CT pulmonary angiography***	***Total***	***Pearson Chi-Square value***	***P value***	***Sig.***
***Sex***	***Female (No/percentage)***	*26 (46.4%)*	*12 (30%)*	*38 (39.6%)*	*1.317*	*0.251*	*NS*
	***Male (No/percentage)***	*30 (53.6%)*	*28 (70%)*	*58 (60.4%)*			
	***Total***	*56*	*40*	*96*			
***The main complaint at the time of CTPA***	***Desaturation***	*10 (17.9%)*	*18 (45%)*	*28 (29.2%)*	*4.160*	0.041*	*S*
	***Hemoptysis***	*2 (3.6%)*	*12 (30%)*	*14 (14.6%)*	*6.542*	0.011*	*S*
	***Progressive dyspnea***	*36 (64.3%)*	*28 (70%)*	*64 (66.7%)*	*0.171*	*0.679*	*NS*
	***Tachycardia***	*4 (7.1%)*	*10 (25%)*	*14 (14.6%)*	*2.987*	*0.084*	*NS*
	***Chest pain***	*10 (17.9%)*	*18 (45%)*	*28 (29.2%)*	*4.160*	0.041*	*S*
***Rising D-dimer level***		*6 (10.7%)*	*32 (80%)*	*38 (39.6%)*	*23.419*	*0.000*^*^*^	*HS*
***NCCT interpretation by RSNA***	***Typical***	*34 (60.7%)*	*22 (55%)*	*56 (58.3%)*	*5.094*	*0.165*	*NS*
	***Indeterminate***	*12 (21.4%)*	*12 (30%)*	*24 (25%)*			
	***Atypical***	*2 (3.6%)*	*6 (15%)*	*8 (8.3%)*			
	***Negative***	*8 (14.3%)*	*0 (0%)*	*8 (8.3%)*			
	***Total***	*56*	*40*	*96*			
***Progressive NCCT***		*4 (7.1%)*	*36 (90%)*	*40 (41.7%)*	*32.953*	*0.000*^*^*^	*HS*

*NCCT* Non-contrast CT

*P value < 0.05 is considered significant

^^^*P* value < 0.01 is considered highly significant

### Regarding the laboratory data

**Fig. 1:**
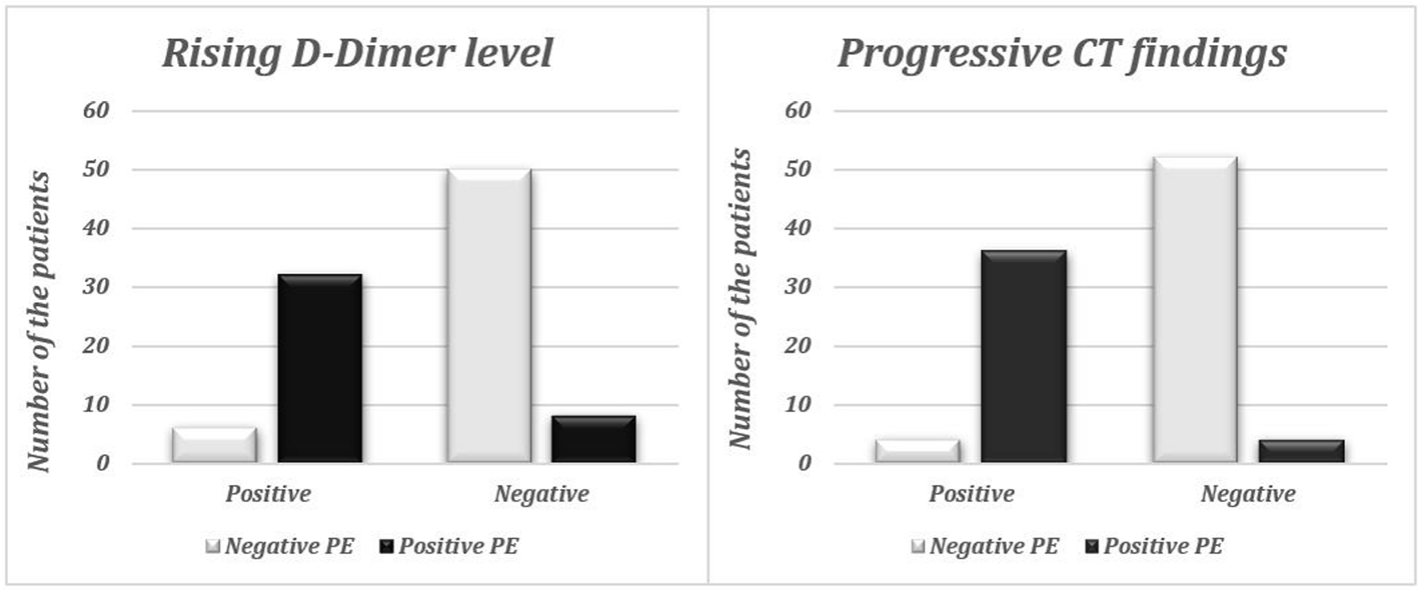
The incidence of rising D-dimer level and the progressive CT findings between the patients with positive and negative pulmonary embolism (PE) according to CTPA interpretation

Patients with positive PE showed higher D-dimer and CRP levels and lower lymphocytic counts compared to the patients with negative PE. However, no significant relation was found between the level of the D-dimer, ferritin, CRP, WBC count, neutrophil, and lymphocyte count and the incidence of PE (Table 2). However, a highly significant correlation was found between the incidence of PE and the rising in the D-dimer level (Table 1, Fig. 1).

**Table 2:** Illustrate the median age, the timing of CTPA, laboratory levels, and CT severity score in correlation with the incidence of pulmonary embolism (PE)

***Wilcoxon rank-sum test***	***CT angiography group***	***Median***	***25 Perc***	***75 Perc***	***Z***	***P value***	***Sig.***
***Age***	***Negative***	*47.5*	*27.5*	*61.75*	*− 1.203*	*0.229*	*NS*
	***Positive***	*55.5*	*39.5*	*67.25*			
***Time for CT angiography***	***Negative***	*9*	*5*	*15.75*	*− 1.09*	*0.276*	*NS*
	***Positive***	*12*	*10*	*16.5*			
***D. dimer level***	***Negative***	*1.26*	*0.575*	*3.2625*	*− 1.119*	*0.263*	*NS*
	***Positive***	*1.8*	*1.185*	*4.425*			
***Ferritin level***	***Negative***	*530*	*198.75*	*815.25*	*− 0.345*	*0.73*	*NS*
	***Positive***	*425*	*270.25*	*701.25*			
***CRP level***	***Negative***	*79*	*12.775*	*167.5*	*− 1.464*	*0.143*	*NS*
	***Positive***	*118.5*	*78.5*	*156.25*			
***WBC count***	***Negative***	*11.2*	*7.6*	*16.425*	*− 0.596*	*0.551*	*NS*
	***Positive***	*10.5*	*8.35*	*13.05*			
***Neutrophil count***	***Negative***	*8.845*	*5.75*	*13.8*	*− 0.282*	*0.778*	*NS*
	***Positive***	*8.4*	*6.425*	*10.15*			
***Lymphocyte count***	***Negative***	*1.44*	*0.76*	*2.205*	*− 0.638*	*0.523*	*NS*
	***Positive***	*1.1*	*0.9125*	*1.575*			
***CT severity score for COVID-19***	***Negative***	*7*	*1.25*	*14.75*	*− 0.326*	*0.745*	*NS*
	***Positive***	*6*	*4*	*10.75*

### Regarding the CT

The median time for CTPA was 9 days for patients with negative PE and 12 days for patients with positive PE with no statistically significant relationship found between the timing of CTPA and incidence of PE (Table 2).

CT with typical criteria for COVID-19 infection was the highest in the two groups yet with no significant relationship found between the COVID-19 probability as reported in CT guided by the RSNA criteria and the incidence of PE (Table 1). The median CT severity score of the COVID-19 was statistically insignificant lower between patients with positive PE (Table 2). However, Progressive CT findings compared to the previous CT showed a highly significant correlation with the incidence of PE (Table 1, Fig. 1).

### In patients with positive PE according to CTPA

**Fig. 2:**
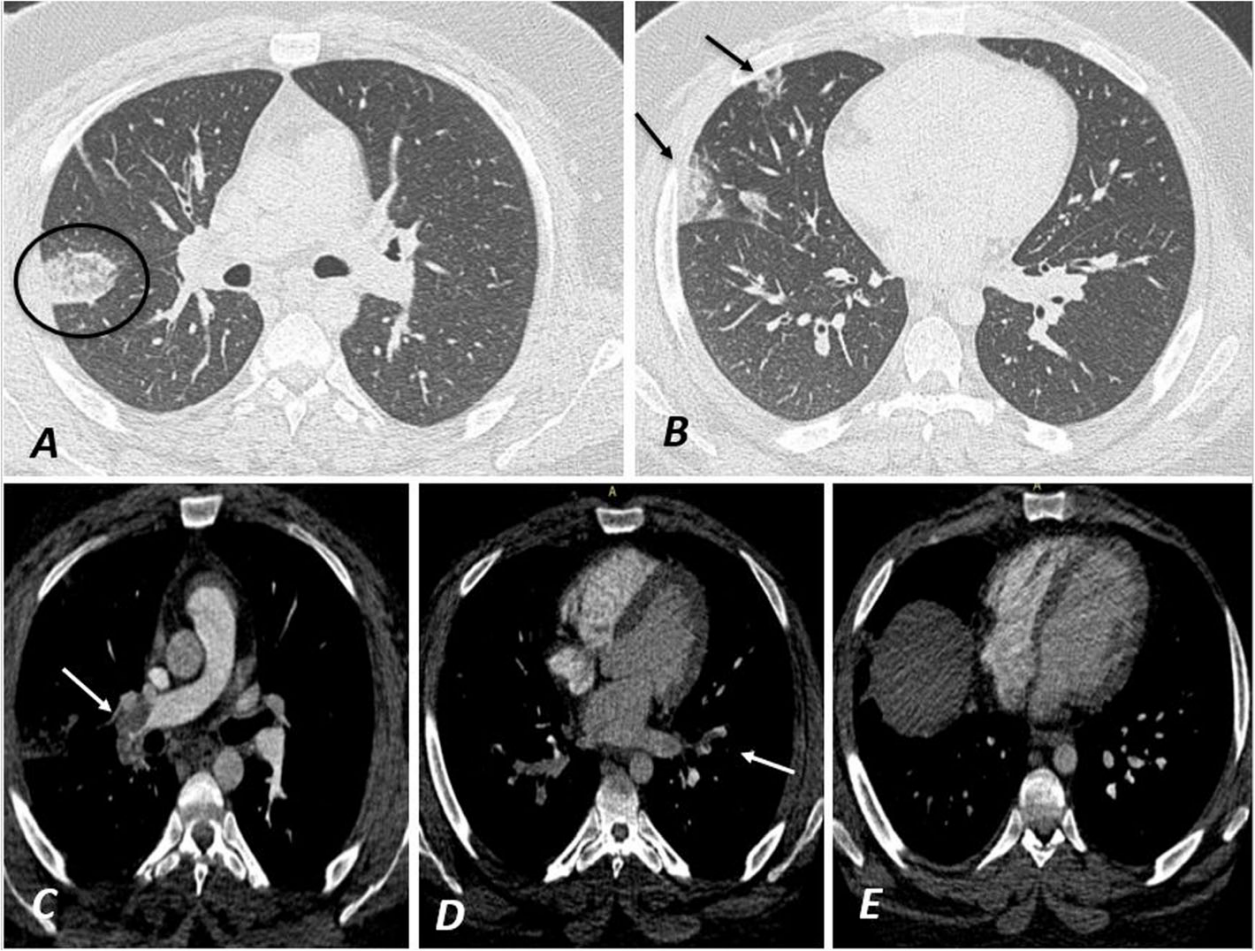
A male patient 52 years old known case of COVID-19 infection as confirmed by PCR. He complained of chest pain 10 days after the onset of the disease. His D-dimer level was 1.6 μg/ml. **a**, **b** Axial CT images of the patient, showing right side peripheral pulmonary reversed halo sign (the black circle in image **a**) with scattered right peripheral pulmonary ground-glass opacities (black arrow in **b**). A pulmonary embolism was suspected for which CTPA was done and revealed pulmonary embolism involving the right inter-lobar artery (white arrow in **c**), partial embolism of the segmental branches of the left lower lobar artery (white arrow in **d**) with no CT evidence of right ventricular strain with normal RV/LV ratio and normal rightward deviated IV septum as seen in image **e**

**Fig. 3:**
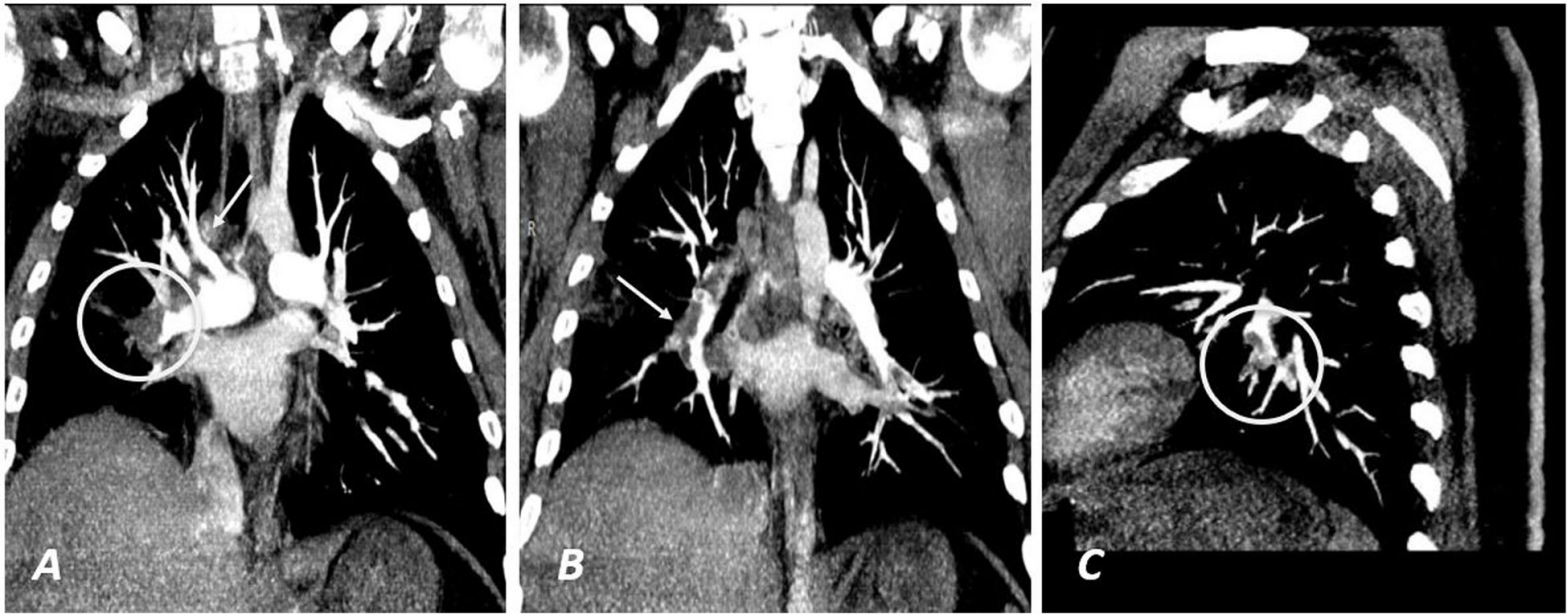
**a**, **b** Coronal maximum intensity projection (MIP) and **c** sagittal MIP images of the same patient in Fig. 2. A thrombus was noted involving the right inter-lobar artery (white circle in **a**) after the origin of the right upper lobar artery (white arrow in **a**) causing its partial occlusion (white arrow in **b**). Another thrombus was noted affecting the segmental branches of the left lower lobar artery (white circle in **c**)

**Fig. 4:**
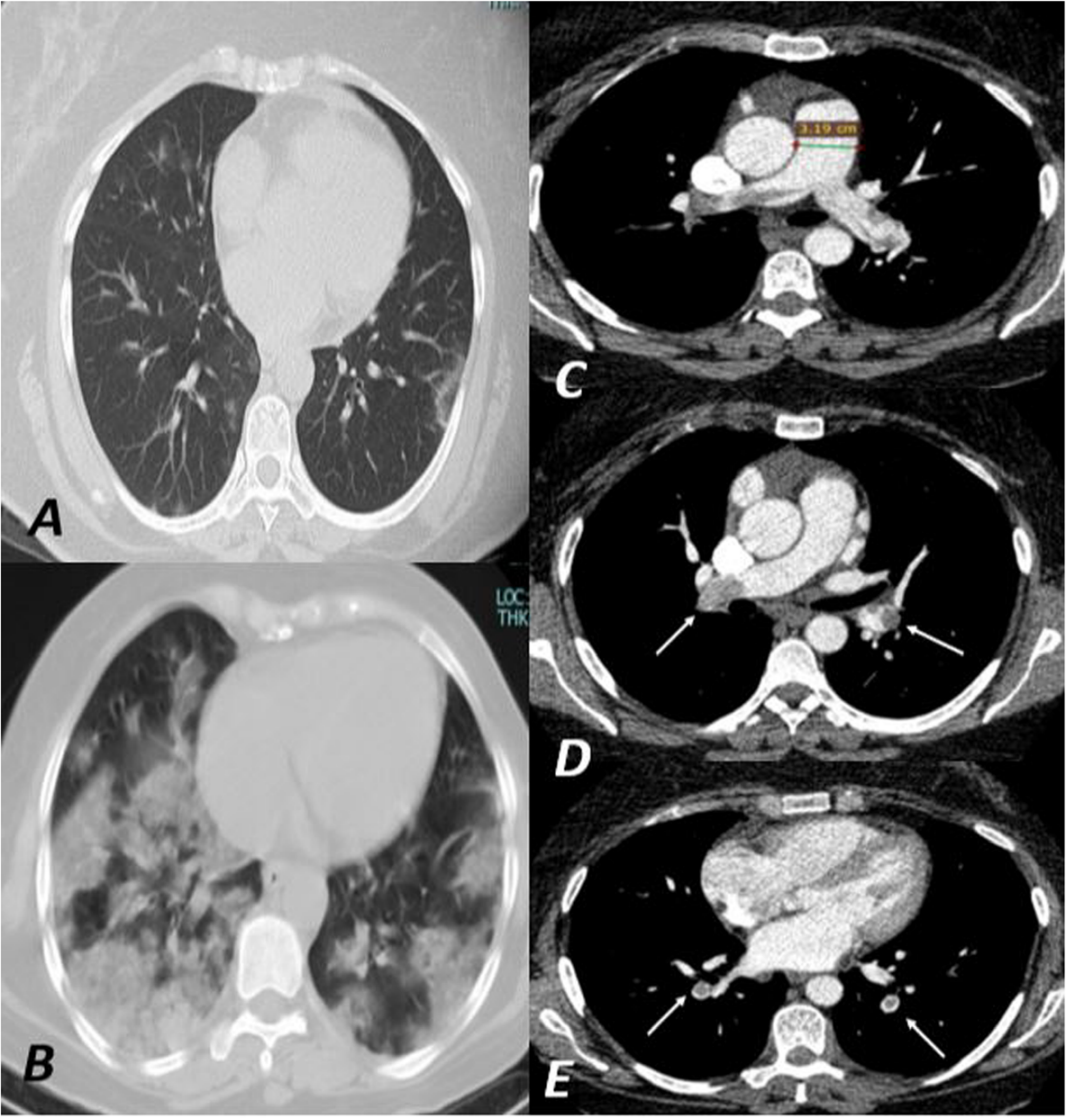
A female patient 48 years old known case of COVID-19 infection as confirmed by PCR. She complained of chest pain 12 days after the onset of the disease and her oxygen saturation level showed a gradual decrease. Her D-dimer level was 1.1 μg/ml and repeated to show a gradual increase reaching 1.8 μg/ml. **a** The axial CT image of the baseline study of the patient showing a few bilateral peripheral patchy areas of ground-glass opacity while **b** was her follow-up after a recent complaint showing a progressive course of the disease extension. A pulmonary embolism was suspected for which CTPA was done and revealed a saddle pulmonary embolism involving the right and left main pulmonary arteries extending into the lobar branches (white arrow in image **d** and **e**). Positive RV strain was identified with dilated main pulmonary artery (31.9 mm in image **c**), reversed RV/LV ratio, and leftward deviation of the IV septum (image **e**)

**Fig. 5:**
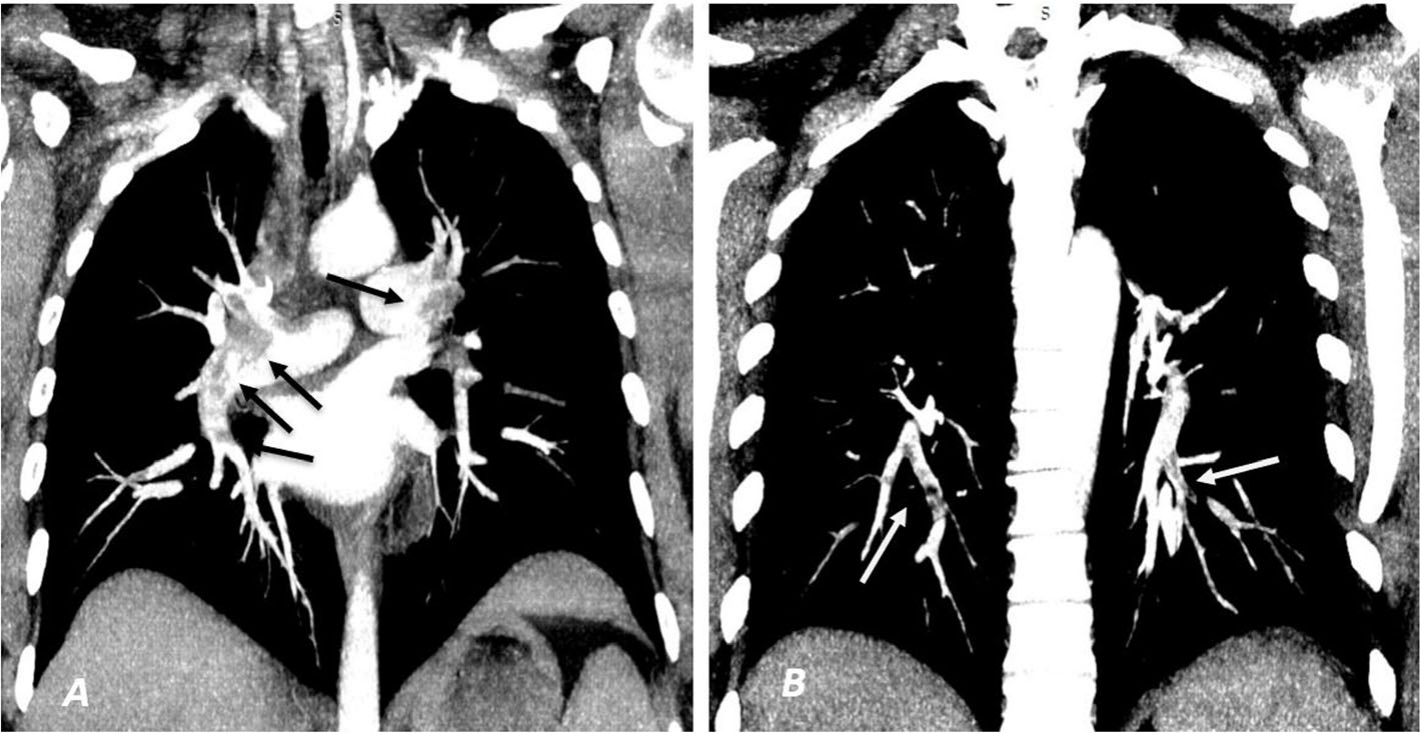
**a**, **b** Coronal maximum intensity projection (MIP) images of the same patient in Fig. 4. A partial thrombus was noted involving the right and left main pulmonary arteries causing partial occlusion extending to involve the related lobar arteries (black arrows in **a**). The thrombus was extending to involve the segmental branches of the lower lobar arteries bilaterally (white arrows in **b**)

Twenty-eight patients representing 70% showed unilateral PE while the rest 12 (30%) of the patients showed bilateral PE. Also, 30 patients (75%) showed PE involving the segmental branches while the rest 10 (25%) showed lobar and segmental branches involvement. Positive RV strain was noted only in 3 cases (7.5%) (Figs. 2, 3, 4, and 5).

## Discussion

The current study focused on highlighting the relationship between the incidence of pulmonary embolism and COVID-19 disease severity either laboratory or by the CT severity score trying to help in early anticipation, diagnosis, and treatment of complicated COVID-19 infection cases.

The study included 96 patients with COVID-19 infection and suspected to have PE, out of which 40 were positive; meaning 41.7% of patients with suspected PE. The most common symptom was worsening dyspnea with an incidence of 66.7% in this study population. However, it was not statistically significant. The statistically significant symptoms were oxygen desaturation, chest pain, and hemoptysis. A similar study by Kaminetzky et al. [8] showed a PE incidence of 37% which is close to our study. This was higher than Poyiadji et al. [5] who found a 22% incidence of PE between their study group, yet they are also similar to our study which concluded no significant difference in age and gender regarding the incidence of PE.

In this article, we studied the relationship between the D-dimer, ferritin, CRP, WBC count, neutrophil, and lymphocyte count and the incidence of PE which was found insignificant although patients with positive PE showed higher levels of D-dimer and CRP compared to the patients with negative PE. Also, the rising D-dimer was found to be highly significant. This mandates incorporation of the follow-up of D-dimer in the management of patients. This could explain the result demonstrated by Kamunetzky et al. [8] that concluded that only a significant indicator was the D-dimer closest to the incidence of PE. Another study by Garcia-Olivé et al. [9] demonstrated that patients with high levels of D-dimer have a higher probability of developing PE. Poyiadji et al. [5] concluded an increase in the D-dimer level of 6 μg/ml had an odds ratio of 2.7 for developing a PE. However, in controversy to our study, they found a significant difference not only with D-dimer but also with CRP. The patients with high D-dimer and high CRP were more susceptible to develop PE. All these inflammatory markers are alarming the use of anticoagulants as advised by many authors [10–12].

The median time to develop PE was found to be 12 days; however, that was statistically insignificant. The duration of illness in our opinion could raise a flag to continuous follow-up of other alarming symptoms and serial measures of D-dimer. The duration in the study done by Garcia-Olivé et al. [9] was 9.7 which is in keeping with our results. This is also similar to Grillet et al. [4] who diagnosed PE at a mean of 12 days from the onset of the symptoms.

The severity of infection detected by CT severity score calculation was found to be not significant with the incidence of PE which is similar to the results concluded by Bompard et al. [1]. However, the progression of the disease was found to be highly significant with the incidence of PE and this was against Bompard et al. [1] who found the higher incidence of CT progression among patients with negative PE. Again, the follow-up of critically ill patients is considered the key to early diagnosis and life-saving for those critically ill patients.

The incidence of segmental PE was higher among our study population with only 3 cases representing 7.5% of the positive cases developed RV strain. This is almost close to the results found by Poyiadji et al. [5] who found more incidence of segmental PE (51% of cases) and the RV strain occurred in 11% of the patients under study.

### Limitation

Our study had few limitations; first, a retrospective study was done in one center, and larger multicentric studies are needed to further understand the nature of this novel complex illness and better manage the second wave. We did not include anticoagulation in our study which will be done in future studies.

## Conclusion

We conclude from our study that for PE to occur during COVID-19 infection, several factors are associated which include disease progression with worsening symptoms as hemoptysis, oxygen desaturation, and chest pain. Rising D-dimer during the second week of disease which all are alert to do CT pulmonary angiography to exclude or confirm PE. This also raises the utmost need for anticoagulation prophylactically.

## Data Availability

Available on request with the corresponding author.

## Abbreviations

COVID-19: Coronavirus disease 2019
CT: Computed tomography
CTPA: CT pulmonary angiography
PCR: Polymerase chain reaction
CRP: C-Reactive protein
PE: Pulmonary embolism
ICU: Intensive care unit
RSNA: Radiological Society of North America
NCCT: Non-contrast CT
WBC: White blood cells
